# Targeted Deletion of *Kcne2* Causes Gastritis Cystica Profunda and Gastric Neoplasia

**DOI:** 10.1371/journal.pone.0011451

**Published:** 2010-07-06

**Authors:** Torsten K. Roepke, Kerry Purtell, Elizabeth C. King, Krista M. D. La Perle, Daniel J. Lerner, Geoffrey W. Abbott

**Affiliations:** 1 Department of Pharmacology, Weill Medical College of Cornell University, New York, New York, United States of America; 2 Department of Medicine, Weill Medical College of Cornell University, New York, New York, United States of America; 3 Department of Veterinary Biosciences, College of Veterinary Medicine, The Ohio State University, Columbus, Ohio, United States of America; The University of Hong Kong, China

## Abstract

Gastric cancer is the second leading cause of cancer death worldwide. Predisposing factors include achlorhydria, *Helicobacter pylori* infection, oxyntic atrophy and TFF2-expressing metaplasia. In parietal cells, apical potassium channels comprising the KCNQ1 α subunit and the KCNE2 β subunit provide a K^+^ efflux current to facilitate gastric acid secretion by the apical H^+^K^+^ATPase. Accordingly, genetic deletion of murine *Kcnq1* or *Kcne2* impairs gastric acid secretion. Other evidence has suggested a role for KCNE2 in human gastric cancer cell proliferation, independent of its role in gastric acidification. Here, we demonstrate that 1-year-old *Kcne2* ^−/−^ mice in a pathogen-free environment all exhibit a severe gastric preneoplastic phenotype comprising gastritis cystica profunda, 6-fold increased stomach mass, increased Ki67 and nuclear Cyclin D1 expression, and TFF2- and cytokeratin 7-expressing metaplasia. Some *Kcne2* ^−/−^mice also exhibited pyloric polypoid adenomas extending into the duodenum, and neoplastic invasion of thin walled vessels in the sub-mucosa. Finally, analysis of human gastric cancer tissue indicated reduced parietal cell KCNE2 expression. Together with previous findings, the results suggest KCNE2 disruption as a possible risk factor for gastric neoplasia.

## Introduction

Gastric cancer remains one of the major causes of mortality, and the second leading cause of cancer death, worldwide. Gastric carcinogenesis is a process that progresses through multiple stages including oxyntic atrophy, ulcer formation, foveolar hyperplasia, hypo- and achlorhydria and mucous cell metaplasia [1]. Despite advances in treatment of gastric cancer, relatively little is known about the molecular mechanisms involved in gastric carcinogenesis, the development of normal gastric mucosa into preneoplastic gastric lesions, or the potential role for ion channels in these processes.

Parietal cells achieve gastric acidification by virtue of an apical H^+^K^+^ATPase (HKA) which pumps protons into the stomach lumen in exchange for K^+^ ions. To maintain this activity, K^+^ ions must travel from the parietal cell into the stomach lumen via the apical membrane, to ensure continued substrate for the HKA [2]. This K^+^ ion efflux occurs through one or more types of apical, K^+^-selective channels. Several inward rectifier (Kir) channels are implicated in this process based on parietal cell expression and pharmacological evidence [3], [4], but thus far there is genetic evidence for just one channel fulfilling this role: KCNE2-KCNQ1 [5]–[7]. KCNQ1 is a six-transmembrane domain α subunit from the S4 superfamily, which forms functional, voltage-gated, homotetrameric K^+^-selective channels in heterologous expression studies [8], [9]. KCNQ1 can also form heteromeric channel complexes with ancillary subunits from the KCNE gene family, and all five known KCNE gene products have been shown to regulate KCNQ1 function in heterologous expression studies [10]. One, KCNE2, converts KCNQ1 to a voltage-independent, constitutively-active channel whose current is increased by low pH. KCNE2 and KCNQ1 are both expressed at or near to the parietal cell apical membrane, and targeted gene deletion of either subunit results in achlorhydria due to impaired gastric acid secretion [5]–[7]. Thus KCNE2-KCNQ1 channels are considered essential for gastric acid secretion and thought to provide luminal K^+^ ions as a substrate for the gastric HKA.

*Kcnq1* ^−/−^ mice and *Kcne2* ^−/−^ mice show similar gastric phenotypes, characterized by achlorhydria, hypergastrinemia and gastric glandular hyperplasia [5]–[7]. Parietal cells from either null show ∼10-fold reduced capacity to recover from proton loading, suggesting a primary defect in gastric acid secretion. *Kcnq1* ^−/−^ mice also develop metaplasia, dysplasia and pre-malignant adenomatous hyperplasia of the stomach independent of infection [11]. This suggests that KCNQ1 dysfunction could lead to gastric neoplasia independent of *H. pylori* due to severe achlorhydria, or represent an additional risk factor which in conjunction with *H. pylori* could predispose to gastric cancer.

The achlorhydria and gastric hyperplasia we previously observed in *Kcne2* ^−/−^ mice was striking given that the pore-forming subunit of the complex, KCNQ1, was still present, and in fact it was strongly expressed in double the number of cells per gastric gland in *Kcne2* ^−/−^ mice compared to *Kcne2* ^+/+^ mice [7]. In a recent study, KCNE2 expression was found to be expressed at relatively low levels in human gastric tumors and in gastric cancer cell lines; furthermore, forced over-expression of KCNE2 suppressed the growth of human gastric cancer cells in tissue culture, and in nude mice [12]. These findings raised the intriguing possibility that KCNE2 might be involved in gastric carcinogenesis, and potentially independently of either *H. pylori* infection or achlorhydria. Here, to further define the potential role of KCNE2 in regulation of normal gastric cell growth, we scrutinized the gastric pathology of *Kcne2* ^−/−^ mice up to 15 months of age.

## Results

### *Kcne2^−/−^* mice exhibit progressive gastric hyperplasia

**Figure 1:**
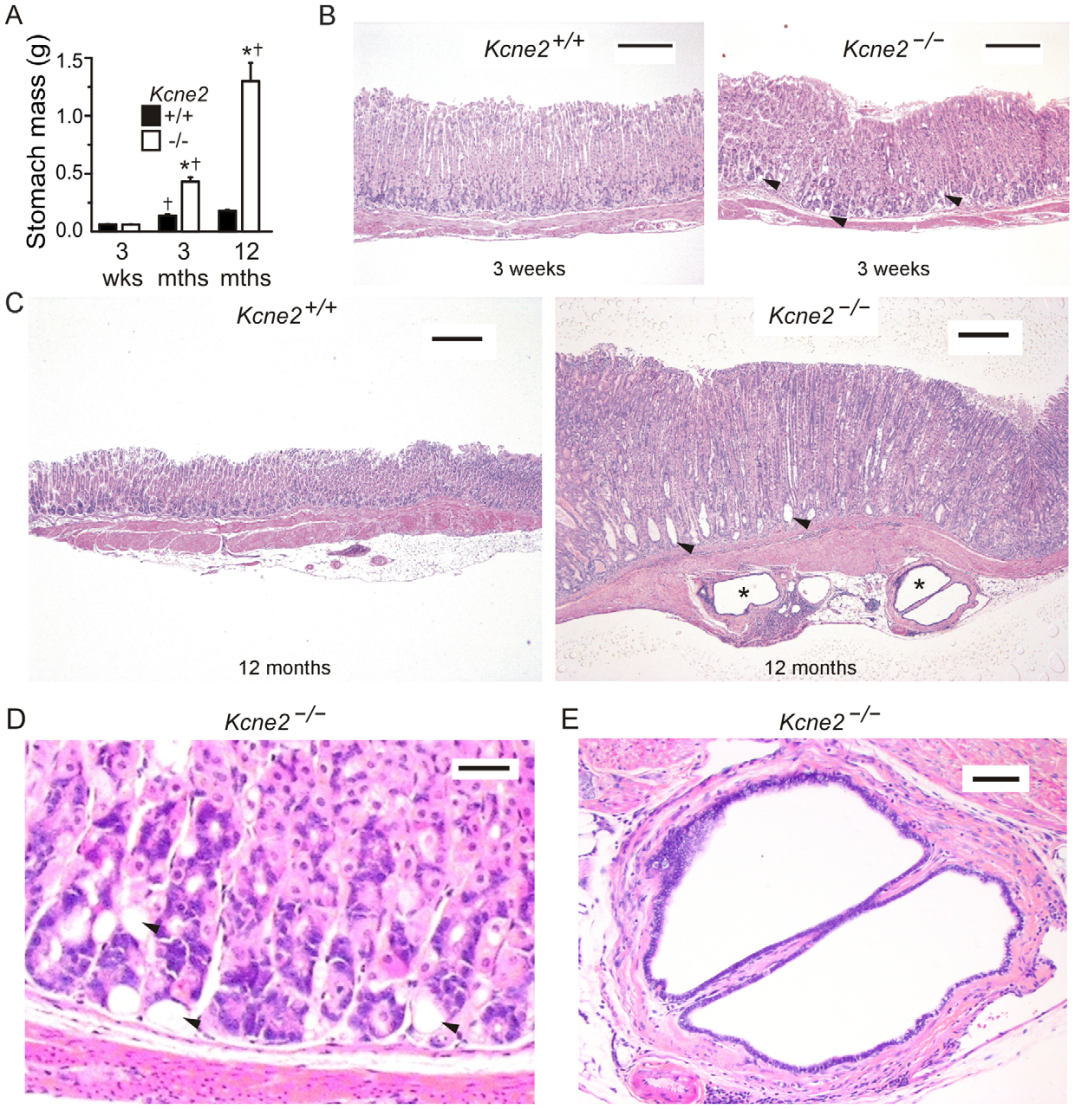
Progression of gastric hyperplasia and GCP in *Kcne2* ^−/−^ mice. A. Mean stomach weights of *Kcne2* ^+/+^ and *Kcne2* ^−/−^ mice at 3 weeks, 3 months and 12 months.^†^significantly different from same genotype at previous time-point, *p*<0.005; * significantly different from *Kcne2* ^+/+^ at same time-point, *p*<0.005; one-way ANOVA. B. H&E-stained sections showing normal gastric mucosa from 3-week-old *Kcne2* ^+/+^ (*left*) and *Kcne2* ^−/−^ (right) mice. Arrows, vacuoles. Scale bar, 200 µm. C. Photomicrograph of H&E-stained sections showing (*left*) normal gastric mucosa from a 12-month-old *Kcne2* ^+/+^ mouse, versus (*right*) gastritis cystica profunda with three herniated glandular profiles in the submucosa in a *Kcne2* ^−/−^ littermate. Arrows, vacuoles. *Cysts. Scale bar, 300 µm. D. Higher magnification of vacuolated region from gastric mucosa of 3-week-old *Kcne2* ^−/−^ mouse from panel B. Arrows, vacuoles. Scale bar, 30 µm. E. Higher magnification of cystic region from gastric mucosa of 1-year-old *Kcne2* ^−/−^ mouse from panel C. Scale bar, 60 µm.

We previously demonstrated that Kcne2 is required for normal regulation of gastric mucosal cell growth: 3-month-old *Kcne2* ^−/−^ mice have gastric hyperplasia, achlorhydria and abnormal parietal cell morphology [7]. Here we compared the stomach mass of 3-week, 3-month- and 12-15-month-old *Kcne2* ^−/−^ mice. The mass of stomachs at 3 weeks of age were similar for *Kcne2^+/+^* and *Kcne2* ^−/−^ mice, whereas by 3 months there was significant gastric hyperplasia in *Kcne2* ^−/−^ mice. This difference had increased by 12–15 months to the extent that *Kcne2* ^−/−^ mice had stomachs 6-fold larger than those of *Kcne2^+/+^* mice of the same age (Figure 1 A).

### *Kcne2^−/−^* mice exhibit gastritis cystic profunda

Even at 3 weeks of age, despite similar stomach mass to that of *Kcne2^+/+^* mice, gastric mucosa of *Kcne2* ^−/−^ mice already showed vacuolations close to the basolateral side (Figure 1 B; magnified in D). To investigate the progression of gastric hyperplasia and its consequences, a total of eleven 12–15 month-old *Kcne2* ^−/−^ mice and five *Kcne2^+/+^* mice were histologically examined. The gastric mucosa in all *Kcne2* ^−/−^ mice showed glandular hypertrophy and diffuse hyperplasia with increased numbers of mucous cells and parietal cells that were occasionally vacuolated (Figure 1 C). Strikingly, gastritis cystica profunda (GCP) was observed in 11/11 *Kcne2* ^−/−^ mice, but 0/5 of the *Kcne2* ^+/+^ mice (χ^2^ *p* = 0.002) (magnification of cyst in Figure 1 E). Dilated glands contained cell debris and, in some but not all *Kcne2* ^−/−^ mice, neutrophils. There was also gastric epithelial hyalinosis with occasional intraluminal crystals, and lymphoplasmacytic aggregates in the mucosa, submucosa and muscularis. In some 12–15-month-old *Kcne2* ^−/−^ mouse stomachs, prominent lamina proprial fibrosis was noted, especially in the superficial layers. GCP, also previously referred to as benign gastric pseudotumor, generally presents as a spectrum of hyperplastic and metaplastic changes following damage to the gastric mucosa. While GCP demonstrates histological features associated with malignancy, it is in itself generally considered functionally benign - although it has been suggested as a possible risk factor for gastric cancer [13], [14].

### Increased proliferative markers in gastric mucosa of *Kcne2^−/−^* mice

**Figure 2:**
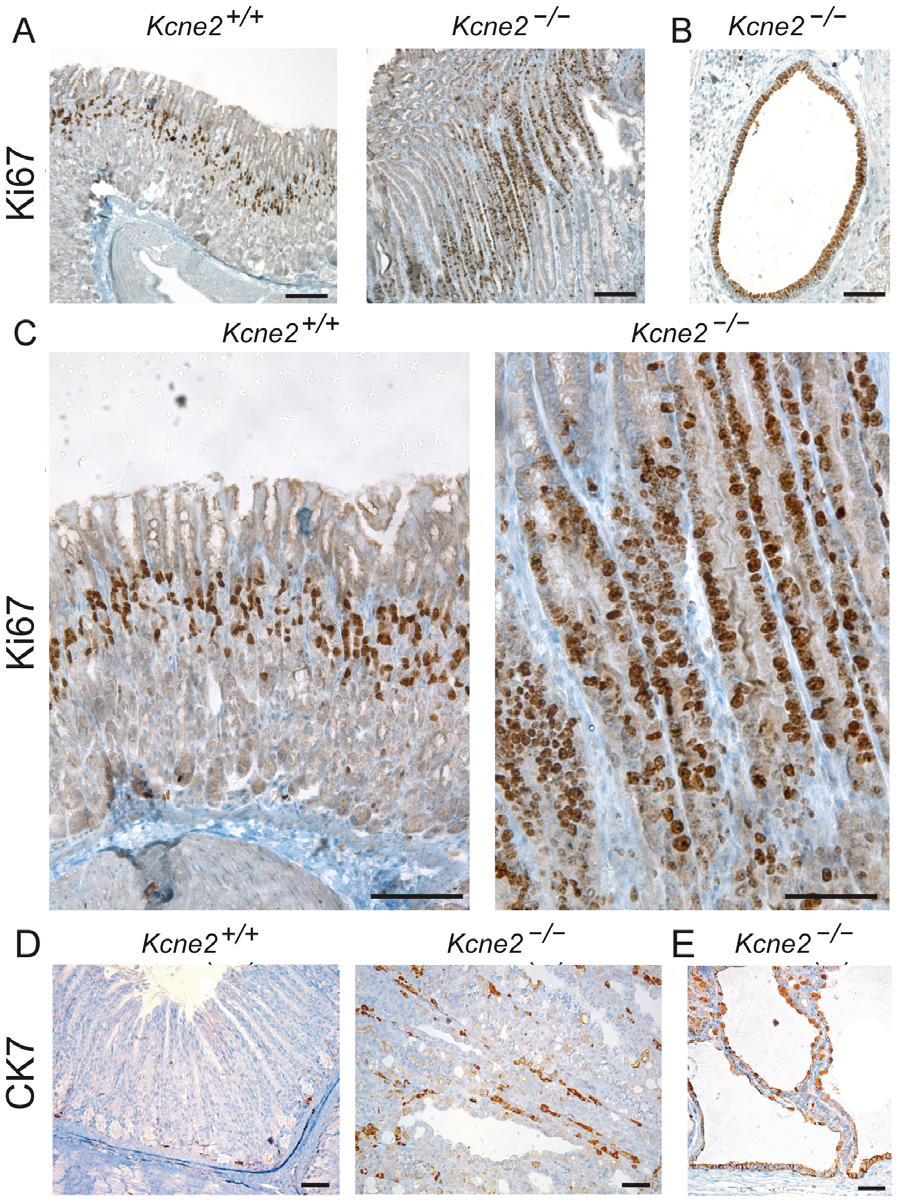
Upregulation of proliferation markers in *Kcne2* ^−/−^ gastric mucosa. A. Immunohistochemical staining for proliferation marker Ki67 indicating an expanded mucosal proliferative compartment in 3-month-old *Kcne2* ^−/−^ compared to *Kcne2* ^+/+^ mice. Scale bar, 200 µm. B. Ki67 staining in an ectopic cystic epithelium in the submucosa of a 12-month-old *Kcne2* ^−/−^ mouse. Scale bar, 50 µm. C. Higher magnification of gastric mucosa from different 3-month-old *Kcne2* ^+/+^ and *Kcne2* ^−/−^ mice from those in panel A, illustrating expansion of proliferative compartment as an increased number of Ki67-positive cells. Scale bar, 200 µm. D. CK-7 staining in longitudinal sections of gastric glands from 12-month-old *Kcne2* ^+/+^ and *Kcne2* ^−/−^ mice. Scale bar, 70 µm. E. CK-7 staining in a cystic complex from a 12-month-old *Kcne2* ^−/−^ mouse stomach. Scale bar, 70 µm.

Importantly, expression of established preneoplastic markers was increased in *Kcne2* ^−/−^ mice compared to *Kcne2* ^+/+^ mice. Ki67 antigen is a cell cycle related nuclear protein commonly used as a proliferation marker in proliferating and neoplastic tissues, including the stomach [15]. Ki67 staining in sections from *Kcne2* ^+/+^ mouse stomachs (3 months) indicated a small band of Ki67 positive cells within the isthmic regions of gastric glands, whereas this proliferative compartment was significantly expanded in age-matched *Kcne2* ^−/−^ mice (Figure 2 A–C). Epithelium lining cystic areas of GCP in 12-month-old *Kcne2* ^−/−^ mice also demonstrated prominent staining of nuclei with Ki67, indicative of a high proliferation rate (Figure 2 B).

Cytokeratin (CK)-7 is a 54 kDa polypeptide expressed in a wide variety of epithelial tissues including lung, breast, and fetal human stomach; however, it is not expressed in normal adult gastrointestinal epithelia [16]. Upregulation of CK-7, indicative of dedifferentiation, was evident in *Kcne2* ^−/−^ gastric mucosa at 12 months; in age-matched *Kcne2* ^+/+^ mice, gastric mucosal positive epithelial cells were much rarer (Figure 2 D). CK-7 was also prominent around cysts (Figure 2 E).

### TFF2-expressing metaplasia in gastric mucosa of *Kcne2^−/−^* mice

**Figure 3:**
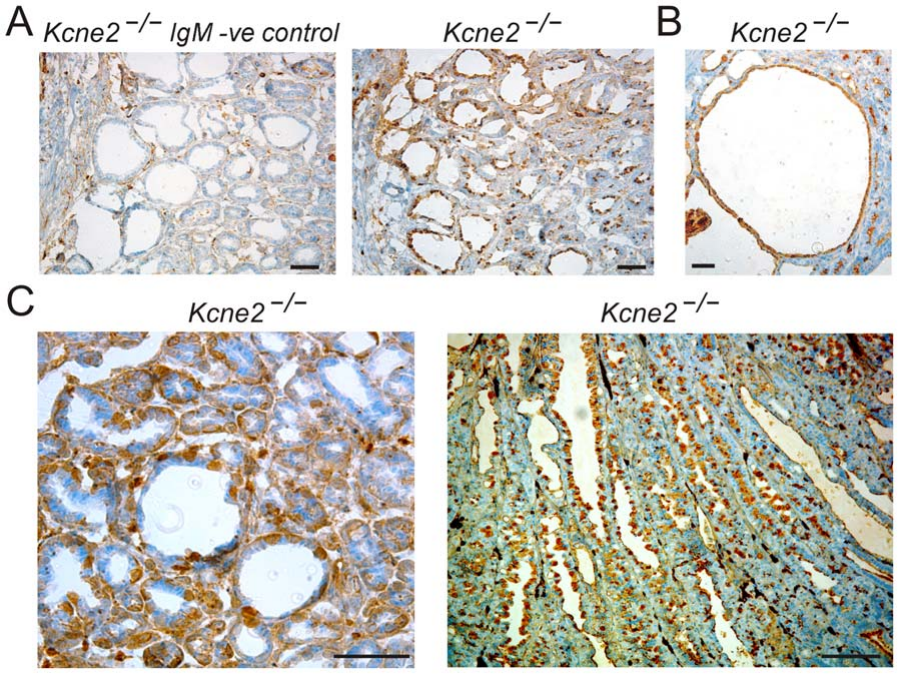
TFF2-expressing metaplasia in *Kcne2* ^−/−^ gastric mucosa. A. *Left*, Negative control for TFF2 staining in areas of GCP from a *Kcne2* ^−/−^ mouse, using IgM isotype. Scale bar, 70 µm. *Right*, TFF2 staining in areas of GCP from a *Kcne2* ^−/−^ mouse, using IgG isotype. Scale bar, 50 µm. B. TFF2 staining in cystic region of gastric mucosa from a *Kcne2* ^−/−^ mouse. Scale bar, 50 µm. C. Increased magnification views of TFF2 staining in gastric mucosa from a *Kcne2* ^−/−^ mouse. *Left*, gastric gland cross-section; *right*, gastric gland longitudinal section. Scale bar, 50 µm.

**Figure 4:**
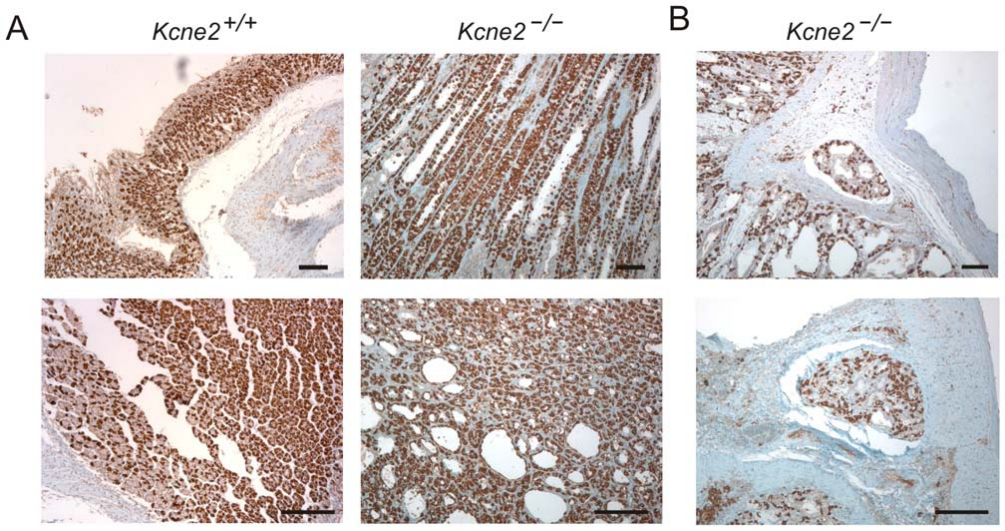
No evidence of reduced parietal cell number in *Kcne2* ^−/−^ gastric mucosa. A. HKA β subunit staining in longitudinal (*upper*) and cross (*lower*) sections from *Kcne2* ^+/+^ and *Kcne2* ^−/−^ gastric glands. Scale bars, 100 µm. B. HKA β subunit staining in cystic regions from a *Kcne2* ^−/−^ mouse. Scale bars, 100 µm.

Trefoil-factor family (TFF)2-expressing metaplasia is associated with progression to gastric cancer in humans, and in Mongolian gerbils infected with *H. pylori* [17]. Gastric mucosa of *Kcne2* ^−/−^ mice exhibited areas of metaplasia with prominent TFF2 expression, including in epithelial cells lining cysts (Figure 3 A–C). TFF2-expressing metaplasia is typically associated with oxyntic atrophy, characterized as a loss of parietal cells [1]. Here, we analyzed gastric mucosa from 12-month-old mice with TFF2-expressing metaplasia for expression of the HKA β subunit (HKA β), a parietal cell marker. Strikingly, there was no evidence of oxyntic atrophy in terms of number of parietal cells (Figure 4 A). Further, HKA β was expressed in cells lining cysts (Figure 4 B).

### Increased gastric mucosal Cyclin D1 expression and gastric neoplasia in *Kcne2^−/−^* mice

**Figure 5:**
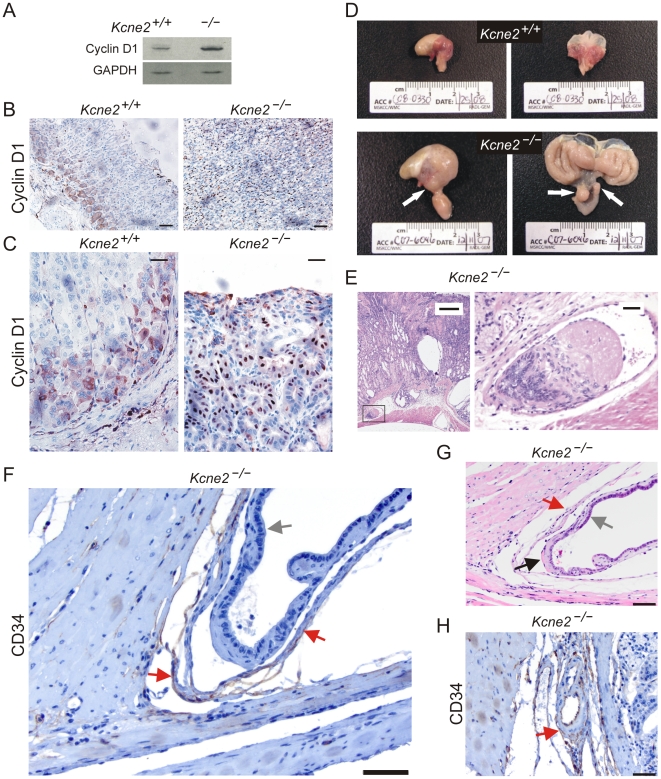
Increased gastric Cyclin D1 expression, and neoplasia, in *Kcne2* ^−/−^ mice. A. Western blots of lysates from 3 stomachs per genotype from *Kcne2* ^−/−^ and *Kcne2* ^+/+^ mice, using anti-Cyclin D1 (upper) and anti-GAPDH (lower) antibodies. B. Cyclin D1 immunoreactivity at the base of *Kcne2* ^+/+^ oxyntic glands (*left*) versus more widespread, and nuclear, staining in the mucosa of *Kcne2* ^−/−^ mice (right). Scale bars: 70 µm. C. Higher magnifications of Cyclin D1 immunohistochemistry panels in B. *Left*, *Kcne2* ^+/+^; *right*, *Kcne2* ^−/−^. Scale bars: 40 µm. D. Serosal (*left*) and mucosal (*right*) views of 12-month-old *Kcne2* ^+/+^ (upper) and *Kcne2* ^−/−^ (lower) mouse stomachs. *Arrows*: *left panel*, cystic changes in the gastric serosa; *right panel*, pyloric polypoid adenomas extending into the duodenum. E. *Left*, H&E-stained section showing a thrombus composed of fibrin and glandular epithelium in a thin-walled vessel within the submucosa of a 12-month-old *Kcne2* ^−/−^ mouse (scale bar, 300 µm). *Right*, expanded view of thrombus from boxed region in left panel (scale bar, 30 µm). F. CD34 staining of endothelial cells in the thin-walled vessel shown in panel E (brown staining, red arrows). Gray arrow, glandular epithelium. Scale bar, 50 µm. G. An H&E-stained section from the thin-walled vessel in panel F, illustrating the vessel wall (red arrow), and invading glandular epithelium (gray arrow) with region of fibrin adhesion (black arrow). Scale bar, 50 µm. H. Internal control for CD34 staining, showing a blood vessel from the same *Kcne2* ^−/−^ mouse stomach as in panels E–G, but without glandular epithelial invasion, stained with anti-CD34 antibody (brown staining, red arrow). Scale bar, 50 µm.

Reduced KCNE2 expression was previously suggested to enhance proliferation in the gastric cancer cell line SGC7901 via increased expression of Cyclin D1 [12]. Here, western blots suggested that *Kcne2* gene deletion increased total Cyclin D1 expression in the gastric mucosa of 12-month-old mice (Figure 5 A). Cyclin D1 showed weak and predominantly cytoplasmic expression at the base of *Kcne2* ^+/+^ oxyntic glands (Figure 5 B,C, left panels), as previously described for normal, proliferating cells in mouse stomach [18]. In contrast, in *Kcne2* ^−/−^ mice, Cyclin D1 showed more widespread and nuclear staining in the neck isthmus region of gastric glands, and glandular pit (Figure 5 B,C, right panels). In two of eleven 1-year-old *Kcne2* ^−/−^ mice analyzed, pyloric polypoid adenomas were observed, extending into the duodenum (Figure 5 D). The same two *Kcne2* ^−/−^ mice also exhibited neoplastic growth in the form of thrombi composed of fibrin and glandular epithelium, in thin-walled vessels (identified using endothelial marker CD34) within the gastric submucosa (Figure 5 E–H).

### Reduced parietal cell KCNE2 expression in human gastric cancer tissue

**Figure 6:**
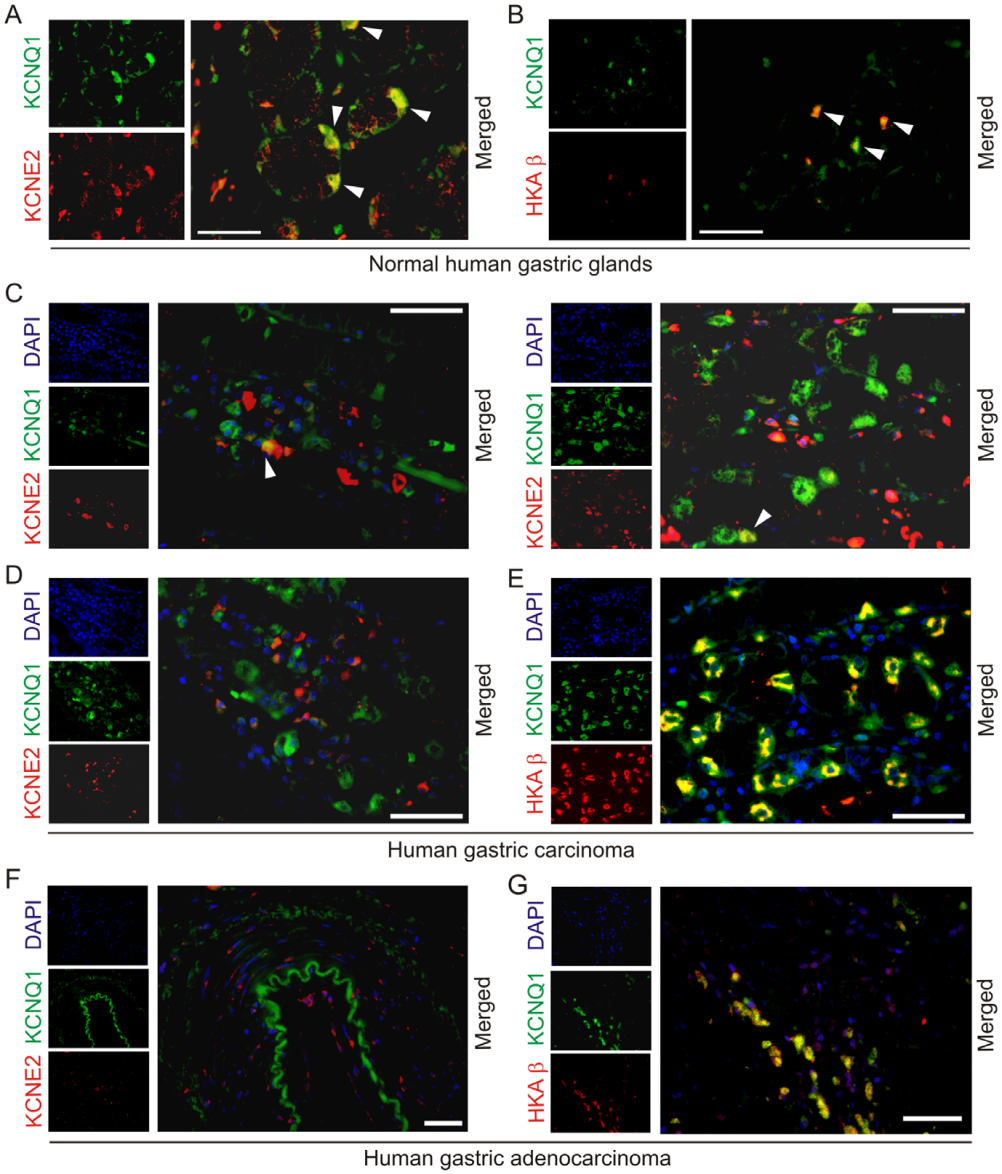
Human parietal cell KCNE2 expression is reduced in gastric carcinomas. A. Immunofluorescence co-labeling of normal (no disease detected) human gastric glands: KCNQ1 (green) and KCNE2 (red). Arrows: co-localization. Scale bar: 100 µm. B. Immunofluorescence co-labeling of normal (no disease detected) human gastric glands: KCNQ1 (green) and HKA β (red). Arrows: co-localization. Scale bar: 100 µm. C, D. Immunofluorescence co-labeling of human gastric glands from an individual with gastric carcinoma: KCNQ1 (green) and KCNE2 (red). Counterstained with DAPI (blue). Scale bar: 100 µm. Arrows: rare co-localization. Panel D shows an example of complete absence of KCNQ1-KCNE2 co-localization. E. Immunofluorescence co-labeling of human gastric glands from an individual with gastric carcinoma: KCNQ1 (green) and HKA β (red). Counterstained with DAPI (blue). Scale bar: 100 µm. Yellow indicates co-localization. F. Immunofluorescence co-labeling of a human gastric adenocarcinoma: KCNQ1 (green) and KCNE2 (red). Counterstained with DAPI (blue). Scale bar: 400 µm. No KCNQ1-KCNE2 co-localization was observed. G. Immunofluorescence co-labeling of a human gastric adenocarcinoma: KCNQ1 (green) and HKA β (red). Counterstained with DAPI (blue). Scale bar: 100 µm. Yellow indicates co-localization.

Given that in a previous study reduced KCNE2 expression was found to enhance gastric cancer cell line proliferation, and KCNE2 expression was found to be reduced in human gastric cancer [12], we examined KCNE2 expression in normal human gastric mucosa, and gastric cancer tissue. Immunofluorescence studies revealed striking differences with respect to KCNE2 and KCNQ1 co-localization, between normal and malignant human gastric mucosae. As expected, KCNQ1 and KCNE2 co-localized strongly in parietal cells in normal human gastric mucosa (Figure 6 A), as did KCNQ1 and HKA β (Figure 6 B). In contrast, in human gastric carcinomas, KCNQ1 and KCNE2 expression rarely overlapped (Figure 6 C, D), even though KCNQ1 retained its strong co-localization with HKA β (Figure 6 E). Thus, KCNE2 expression in parietal cells was rarely observed in gastric carcinoma. The lack of KCNE2-KCNQ1 co-localization was even more profound in human gastric adenocarcinoma (Figure 6 F), which again retained co-localization of KCNQ1 and HKA β (Figure 6 G).

## Discussion

### Potassium channels in proliferative disorders

Potassium channels have emerged as a potential target for anti-cancer therapies [19], including KCNQ1, hERG and Kv2.1, all of which are α subunit partners of KCNE2. Disrupted imprinting caused by mutations in the KCNQ1 gene cause Beckwith-Wiedemann syndrome (BWS), which predisposes to cancer [20]–[22], and KCNQ1 knockout predisposes to gastric metaplasia [11]. The hERG potassium channel α subunit, which forms complexes with the KCNE2 ancillary subunit in human heart [23], is over-expressed in some tumors, and has been identified as a tumor survival factor [24]–[27] as has Kv2.1 [28].

KCNE2 was previously found to be expressed 2-fold lower in human gastric cancer tissue than in neighboring normal gastric cells, and forced upregulation of KCNE2 had anti-proliferative effects on gastric cancer cells *in vitro* and injected into nude mice [12]. The postulated mechanism for this anti-proliferative effect is down-regulation of Cyclin D1, delaying progression through the cell cycle [12] similar to what was observed with the hERG blocker cisapride [29]. Elevated expression of Cyclin D1 in human gastric tumors correlates with a particularly poor prognosis [30], therefore understanding factors that increase Cyclin D1 expression may lead to therapeutic avenues for this and other forms of cancer. Overexpression of Cyclin D1 has been suggested to contribute to oncogenesis by disturbing the cell cycle, and has been reported to be an important oncogenic factor in esophageal carcinoma [31], and associated with nuclear accumulation of β-catenin in ovarian endometrioid adenocarcinomas [32]; nuclear Cyclin D1 overexpression in gallbladder carcinomas is a critical event [33].

Here, we describe extensive metaplastic changes in the gastric muscosa due to genetic disruption of *Kcne2*. This is associated with increased nuclear Cyclin D1 expression in the gastric mucosa, reminiscent of results from previous *in vitro* studies of KCNE2 [12]. Neither the current report nor the previous study, however, delineate the mechanism for Cyclin D1 upregulation in *Kcne2* ^−/−^ mucosa – is it a consequence of metaplastic changes secondary to achlorhydria in these mice, or is there a more direct association between *Kcne2* and Cyclin D1? The study by Yanglin and colleagues argues for the latter at least in part, because in their study the Cyclin D1 changes occurred in isolated cells without the influence of achlorhydria [12]. We consider that changes secondary to achlorhydria are the most likely dominant factor, but a direct link cannot be excluded. Interestingly, hERG, another partner of KCNE2, is expressed in gastric cancer cells but not normal gastric epithelia, and the hERG channel blocker cisapride was previously found to suppress gastric cancer cell growth by inhibiting entry into S phase from G(1) phase in the cell cycle, increasing apoptosis [29]. Because KCNE2 partially suppresses hERG currents by reducing unitary conductance and speeding deactivation [23], it is an intriguing possibility that the observed anti-proliferative effects of KCNE2 overexpression *in vitro* [12] are due to hERG current suppression promoting apoptosis, and conversely that KCNE2 down-regulation or genetic disruption may favor gastric cellular proliferation by increasing hERG current density.

### GCP and TFF2-expressing metaplasia (SPEM) in *Kcne2^−/−^* mice

GCP can present in human beings with intermittent epigastric pain, bloating, gastric obstruction or upper gastrointestinal bleeding, and is most commonly seen in patients with a history of either gastrectomy or gastrostomy [34]. However, a few cases have been reported with no association to prior gastric surgery [35], [36]. The pathogenesis of human GCP is believed to arise from an injury of the muscularis mucosae, which may lead to ectopic entrapping of gastric glands in the submucosa, muscularis mucosae or serosa, or from chronic inflammation. GCP is commonly considered a benign clinical entity, although association with gastric cancer has been reported [14], [37], [38].

In laboratory animals, GCP secondary to *Helicobacter sp*. infection has been described as a predecessor of intramucosal dysplasia and neoplasia [39], and associated with development of gastric carcinoma. GCP was frequently found in *H. pylori*-infected Mongolian gerbils that also developed gastric adenocarcinoma and lymphoid hyperplasia [40]. In a previous murine genetic model of GCP, TGF beta 1^+/−^ mice developed glandular hyperplastic lesions that shared morphologic features with human GCP, with mixed inflammatory infiltration of the surrounding mucosa and chronic vasculitis in the tissues adjacent to these lesions [41]. Here, in *Kcne2* ^−/−^ mice, GCP occurs in the absence of a primary inflammatory lesion, and independent of vascular obliteration. Instead, we propose that the primary defect is loss of the KCNE2 β subunit from channel complexes with KCNQ1, thereby impairing an important apical K^+^ recycling pathway in parietal cells and preventing gastric acidification by the parietal cell HKA. While this represents a ‘functional atrophy’ in the parietal cell population, in previous studies of intestinal metaplasia an actual loss of parietal cells, termed ‘oxyntic atrophy’, was considered an important process in the etiology of intestinal metaplasia, associated with acid reflux disease or *H. pylori* infection, and is the most common metaplastic process observed in the upper gastrointestinal tract [42].

Oxyntic atrophy in the absence of significant inflammation can be induced by DMP-777, a neutrophil elastase inhibitor which also targets parietal cells due to its action as a protonophore [43]. Oxyntic atrophy in gastric-deficient mice, following treatment with DMP-777, leads rapidly to TFF2-expressing metaplasia, also referred to as spasmolytic peptide expressing metaplasia (SPEM) [44]. SPEM is notable because of its strong association with human gastric adenocarcinoma, and it is observed in the majority of fundic gland biopsies from human *H. pylori*-infected gastritis patients [45]. The appearance of SPEM in *Kcne2* ^−/−^ mice in the absence of either *H. pylori* or classic oxyntic atrophy suggests that the ‘functional atrophy’ caused by *Kcne2* deletion may be enough to trigger SPEM. One major difference between *Kcne2* ^−/−^ mice and those in the Nozaki study is that *Kcne2* ^−/−^ mice are hypergastrinemic, not gastrin-deficient. Another difference is that DMP-777 disrupts a gastric tubulovesicle proton gradient without impairing H^+^K^+^-ATPase function, whereas targeted *Kcne2* disruption prevents H^+^/K^+^-ATPase function by removing its luminal substrate, K^+^ ions [7], [44]. Perhaps this latter mechanism may in the future reveal clues regarding the critical events in oxyntic atrophy that result in SPEM: it is currently suggested that SPEM induction results from a deficiency in parietal cell-secreted regulators of normal gastric mucosal renewal, which include sonic hedgehog and TGF-α [46], [47] due to loss of parietal cells. Further studies of *Kcne2* ^−/−^ parietal cells - which are still present but malfunctioning - may reveal regulators which they can still secrete, and those which they cannot.

The gastric mucosa of *Kcne2* ^−/−^ mice also show increased expression of the established proliferation marker Ki67, manifesting as an increase in the width of the mucosal proliferative band, and as Ki67-positive cells lining the cysts in regions of GCP. Similarly, the de-differentiation marker CK7, not expressed in 1-year-old *Kcne2* ^+/+^ gastric mucosa, was widely expressed in that of *Kcne2* ^−/−^ mice. Taking these and the markers described above together, the *Kcne2* ^−/−^ gastric mucosa exhibits metaplasia with multiple features of preneoplasia, and in some cases neoplasia, notably in a specific pathogen-free environment with no evidence of gastric *Helicobacter* infection or oxyntic atrophy, and in the absence of chemical inhibitors of gastric acidification. Together with the finding here that parietal cell KCNE2 expression appears to be reduced in human gastric cancer tissue (Figure 4) and the previous report that KCNE2 inhibits gastric cancer cell proliferation [12], the data suggest KCNE2 disruption is associated with gastric cancer progression. Future studies will involve examining whether *Kcne2* disruption increases predisposition to gastric cancer within pathogen and carcinogen-based protocols, the mechanisms behind these possible differences, and the potential mechanistic links between *Kcne2*, Cyclin D1 and cell cycle perturbation outside the realm of achlorhydria-associated disease etiology. Furthermore, as KCNQ1 and KCNE2 are also co-expressed in thyroid epithelial cells, where they are important for thyroid hormone biosynthesis [48], it will be of interest to examine a potential role for KCNE2 in abnormal thyrocyte proliferation.

## Materials and Methods

### Generation and use of gene-targeted mice

All mice described in this study were housed, utilized and euthanized according to NIH and Cornell University Institutional Animal Care and Use Committee guidelines. All mice described in this study were housed, utilized and euthanized according to NIH and Weill Medical College Institutional Animal Care and Use Committee guidelines. Ethical approval to breed and harvest tissue from wild-type and *Kcne2* ^−/−^ mice for biomedical research was approved by Weill Medical College Institutional Animal Care and Use Committee (protocol 0704-610A). *Kcne2* ^−/−^ mice were generated as previously described from C57BL/6 *Kcne2^+/^* ^−^ x *Kcne2^+/−^* crosses [7]. Numerical data were analyzed with EXCEL software (Microsoft) using one-way analysis of variance (ANOVA) with statistical significance set at *P*<0.05.

### Histology

For histology and stomach mass quantification, *Kcne2^+/+^* and *Kcne2* ^−/−^ mice at 3 weeks, 3 months and 12–15 months were sacrificed using CO_2_ asphyxiation (5–10 per genotype). Stomachs were removed post-mortem, stomach mass determined, then stomach tissue was fixed in 10% neutral buffered formalin, processed by routine methods and embedded in paraffin wax. Gastric mucosal sections were cut at 5 µm intervals, placed on positively-charged Superfrost slides, stained with hematoxylin and eosin (H&E), and evaluated with an Olympus BX45 microscope.

### Immunohistochemistry

Immunohistochemical detection of Ki67, CK-7 and TFF2 (also known as spasmolytic peptide) was performed using a Discovery XT processor (Ventana Medical Systems). The primary antibody concentrations used were: 0.05 µg/ml (rabbit polyclonal anti-Ki67, Vector Labs); 1 µg/ml (mouse monoclonal anti-CK-7; Abcam); 1 µg/ml (mouse monoclonal anti-TFF2; Abcam). Preceding primary antibody incubation, tissue sections were blocked for 30 min in 10% normal goat serum, 2% BSA in PBS (anti-Ki67); 30 min in 10% normal goat serum, 2% BSA in PBS and Avidin/Biotin for 8 min (anti-CK-7); 30 min in 10% normal goat serum, 2% BSA in PBS and Avidin/Biotin for 4 min (anti-TFF2). Primary antibody incubation times were: 3 hr (Ki67 and CK-7); 5 hours (anti-TFF2). Secondary antibody incubations were: 32 min in 1∶200 biotinylated goat anti-rabbit IgG (Vector Labs) for Ki67; 60 min in 1∶200 biotinylated horse anti-mouse IgG (Vector Labs) for CK-7 and TFF2. For Ki67 secondary antibody incubation, Blocker D, Streptavidin-HRP and DAB detection kit (Ventana Medical Systems) were used according to the manufacturer's instructions. For CK-7 and TFF2, mouse IgG1 (5 µg/ml) was used as an isotype negative control, and Blocker D, Streptavidin-HRP and DAB detection kit (Ventana Medical Systems) were used.

For Cyclin D1 detection, stomach slides were heated at 58–60°C for 30 min and then deparaffinized. Endogenous peroxidase activity was blocked by incubating sections in 1% hydrogen peroxide in PBS for 15 min (8.3 ml 30% H_2_O_2_/241.7 ml PBS) followed by unmasking of the antigenic epitope by microwaving in 10 mM Citrate Buffer at high power for 15 min. Slides were cooled for 20 min, washed in distilled water for 5 min, transferred to PBS and incubated in 10% normal horse serum (in 2% BSA-PBS diluent) for 30 min in a humid chamber. Mouse monoclonal anti-cyclin D1-antibody (Cell Signaling) was added at 1∶1000 with 2% BSA in PBS and incubated overnight at 4°C in a humid chamber. Slides were rinsed in PBS and the secondary biotinylated horse anti-mouse IgG antibody (Vector Labs) was applied at 1∶500 in PBS for 30 min at RT. Slides were washed and an Avidin-Biotin Complex (Vectastain ABC Elite Kit, Vector Labs) diluted at 1∶25 in PBS was applied for 30 min. The signal was detected by incubation in 3,3-Diaminobenzidine (Sigma, Batch No. 026K3767) until the desired color intensity was reached. After final washes in water, slides were lightly counterstained in hematoxylin. KCNQ1 and HKA β single labeling and detection were performed as previously described [7]. CD34 detection was with 1∶50 anti-CD34 antibody (AbCam). Slides were viewed with a Nikon Eclipse E600 microscope and photographed using a RT Color Camera and SPOT software (Diagnostic Instruments, Inc.).

### Immunofluorescence

All human tissue was obtained from ProSci, Poway, CA, and was certified as having been obtained from qualified and licensed pathology services, using appropriate conformed consent for use in biomedical research and with donor anonymity protection; therefore, additional Weill Medical College Institutional Review Board ethics approval was not required. Immunofluorescence detection of HKA β, KCNQ1 and KCNE2 in human gastric tissue (ProSci) was performed using a Discovery XT processor (Ventana Medical Systems). The following human tissues were used: normal stomach tissue from a 50-year-old woman (PSC-10-809-XB1); gastric carcinoma tissue, patient information unavailable (PSC-10-814-CA1); gastric adenocarcinoma tissue from a 51-year-old woman (PSC-10-809-XA1). The primary antibody concentrations used were: 0.5 mg/ml anti- HKA β (mouse monoclonal, Affinity Bioreagents), 1 mg/ml anti-KCNQ1 (rabbit or goat polyclonal, Chemicon), and in-house anti-KCNE2 serum was used at a 1∶500 dilution after column-enriching IgG. Preceding the primary antibody incubation, the tissue sections were blocked for 30 min in 10% normal goat serum, 2% BSA in PBS, followed by 8 min Avidin/Biotin block. The primary antibody incubation (3 hr) was followed by 32 min incubation with biotinylated anti-mouse IgG (ABC kit from Vector labs), 60 min incubation with biotinylated anti-goat IgG (ABC kit from Vector labs), or biotinylated anti-rabbit antibody at 1∶200 dilution (Vectastain ABC kit). The secondary detection was performed with Streptavidin-HRP D (Ventana Medical Systems), followed by incubation with Tyramide-Alexa Fluor 488 (Invitrogen) or Tyramide Alexa Fluor 568 (Invitrogen). Stained slides were viewed with a Zeiss Axiovert 200 widefield microscope and pictures were acquired using MetaMorph software 7.1 (Molecular Devices).

### Western blotting

For western blotting, gastric membrane fractions were prepared as previously described [7]. Three stomachs from 12-month-old *Kcne2^+/+^* and *Kcne2* ^−/−^ mice were removed post-mortem, cut open along the curvatura ventriculi major, cleared of ingesta and immediately snap-frozen in liquid nitrogen. Stomachs were then homogenized in a buffer containing 50 mM Tris-HCl, 150 mM NaCl, 100 ug/ml PMSF, 1 ug/ml Nonidet P40, 0.5% SDS and 0.5% sodium orthovanadate, then incubated on ice for 30 min and centrifuged at 12000×*g* for 20 min at 4°C. Protein concentration of the supernatant was measured according to the Bradford method. Total protein (40 µg/lane) was loaded into a pre-cast tris-glycine 4–20% gel (Jule Inc, Milford, CT) and separated by electrophoresis. Proteins were then transferred onto a PVDF membrane (Bio-Rad, Hercules, CA), and blocked with 5% milk, 0.05% Tween-20 in PBS for 12 hours at 4°C on a rocker. Primary antibody incubations (4 hr, RT in 1% milk, 0.05% Tween-20, PBS, ‘Buffer A’) were: 1∶2000 anti-Cyclin-D1 (Abcam); 1∶5000 anti-GAPDH (Abcam). Membranes were washed 4 times, 20 min each with antibody incubation buffer then incubated with the appropriate secondary antibodies (BioRad) diluted 1∶10000 in Buffer A for 2 hr at RT then washed 4×20 min each with Buffer A and once for 5 min with PBS. Membranes were incubated for 1 min with the SuperSignal ECL reagent (*Pierce*) then exposed on BioMax Light Film (Kodak) and developed using an RP X-OMAT Processor (Kodak).
